# Reconstruction of the pelvic ring using an autologous free non-vascularized fibula graft in a patient with benign fibrous histiocytoma

**DOI:** 10.1186/1477-7819-2-38

**Published:** 2004-11-23

**Authors:** Philipp Niemeyer, Karl Ludwig, Mathias Werner, Ludger Bernd, Dominik Parsch

**Affiliations:** 1 Orthopedic University Hospital, Heidelberg, Germany; 2 Institute of Osteopathology, University Hospital Hamburg, Germany

## Abstract

**Background:**

Benign fibrous histiocytomas (BFH) usually presents as a small benign lesion that predominantly occurs in the skin. Only few cases of BFH arising from bone have been reported, its occurrence in pelvic bones is even rarer.

**Case presentation:**

A 34-year-old female presented with BFH at a rare anatomical location on both sides of the *os ilium *which was larger than earlier reported BFH of the bone. Surgical resection was performed successfully including resection of the inner pelvic ring and reconstruction of the linea terminalis using a non-vascularized fibular autograft. At 18 months after tumor resection and reconstruction of the pelvic ring, with interposition of a free vascularized fibula graft patient has an excellent clinical oncological and functional outcome.

**Conclusion:**

Non vascularized fibular autograft is a useful reconstructive procedure in select patients.

## Background

Benign fibrous histiocytoma (BFH) is a tumor that occurs predominantly in the skin (also called dermatofibroma) and most commonly in younger individuals. The tumor typically presents as a painless nodule varying in size from a few millimeters to several centimeters.

BFH of the bone has been a subject of increasing interest within the past few years. The term was initially introduced by Dominok in 1980 to describe a cystic lesion in the femur of a 66-year-old man [1]. Only a few cases of BFH of bone have been described in the literature since then, and even fewer located in the pelvis [2-6].

Histologically, BFH arising from soft tissues cannot be distinguished from those arising from bone [7]. Pain is most often the predominant presenting symptom [7], which helps to distinguish this tumor clinically from other fibrous lesions, such as non-ossifying fibroma. Peak incidence of BFH arising from bone is reported in the third decade [5].

On radiography the lesion is characterized by osteolytic lesions with a well defined sclerotic margin [6]. Magnetic resonance (MR) Imaging of BFH usually shows low signal intensity on T1- and high signal intensity on T2-weighted images. Peripheral contrast enhancement has also been described in BFH [4,8,9]. Computed tomographic (CT) scan typically show fibrous osteolytic lesions with cortical thinning [10]. Positive bone scans may be helpful in differentiate BFH from nonossifying fibroma [4]. Biopsy is mandatory to confirm the diagnosis: Histologically BFH represents a benign but diverse group of neoplasms that are characterized by both fibroblastic and histiocytic differentiation. Giant cells of the "Touton" type and a "storiform pattern" are typically seen [11]. BFH shows proliferation of benign oval or spindle cells resembling fibroblasts mingled with cells resembling histiocytes. Foam cells are a prominent component of the macroscopically yellow zones of the tumor. It may be difficult to differentiate BFH from low-grade malignant fibrous histiocytoma. Lack of marked pleomorphism or of atypical mitoses is suggestive of a benign diagnosis. On microscopic examination some forms of BFH show features of dysplasia, such as hypercellularity, mitotic activity or focal necrosis [2]. These entities are considered to be more aggressive types of BFH, that should be treated with wide surgical excision [2]; for non-aggressive types of BFH even intralesional resection can be effective [7].

A local recurrence rate of 5 to 25 % is reported in literature. The recurrence rate is typically related to the size of the tumor [2,12]. Consequently, careful clinical and radiological follow-up including regular MRI is recommended [2]. We present here a case of BFH treated with resection and reconstructed using non vascularized fibular bone graft.

## Case presentation

**Figure 1:**
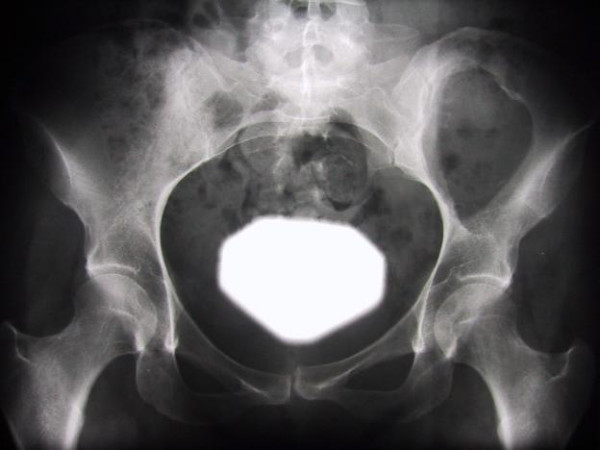
Radiograph of the pelvis showing a well-circumscribed osteolytic lesion in the left iliac bone; it is sharply marginated with a thin sclerotic rim and without any matrix calcifications.

**Figure 2:**
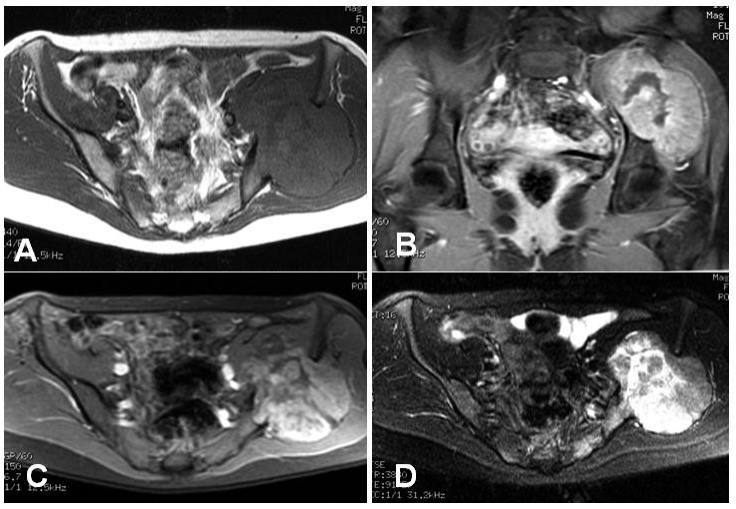
a-d: MRI shows a mass originating from the ileum and extending into the soft tissues both anteromedially and posterolaterally. In T_1_-weighted sequences the mass is isointense with muscle (a). It enhances after administration of gadolinium (b, c). In fat-suppressed T_2_-weighted sequences it has high signal intensity (d).

A 34-year-old female presented with a 3 year history of a palpable swelling and mild pain in the left gluteal region. There was no history of preceding trauma or accident. Two years after initiation of symptoms without clinical progress, the patient became pregnant and noted a local growth of the mass. After giving birth to a healthy child, the patient consulted an orthopedic surgeon. A radiographic image of the pelvis revealed a large osteolytic lesion in the left iliac wing, sharply contoured with sclerotic margins, indicating a slow growth pattern (Figure 1). MR-imaging of the pelvis showed a mass originating from the os *ilium *with a large extra- and intrapelvic soft-tissue component (Figure 2). Open biopsy was performed that lead to the diagnosis of BFH.

**Figure 3:**
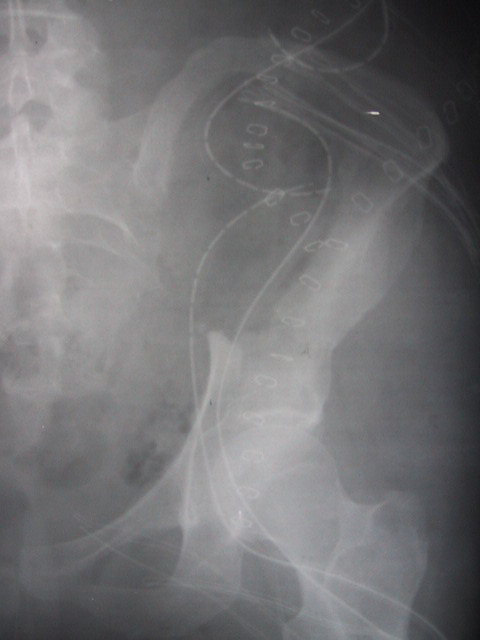
Defect after tumor resection, with disruption of the linea terminalis.

**Figure 4:**
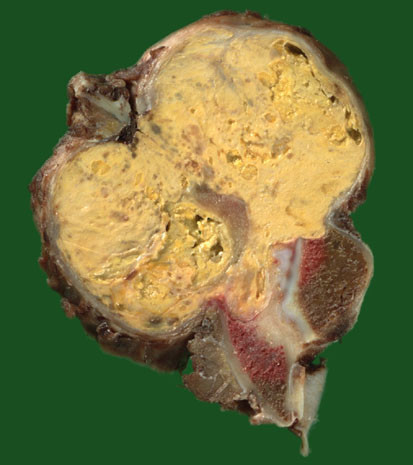
Macroscopic view of the resection specimen in transverse orientation. Large yellow osteodestructive tumor originating from iliac bone and with extensive extraosseous parts. Invasion of massa lateralis of sacral bone. Sharp borders between tumor and bone are indicative of a slow-growing neoplasm.

**Figure 5:**
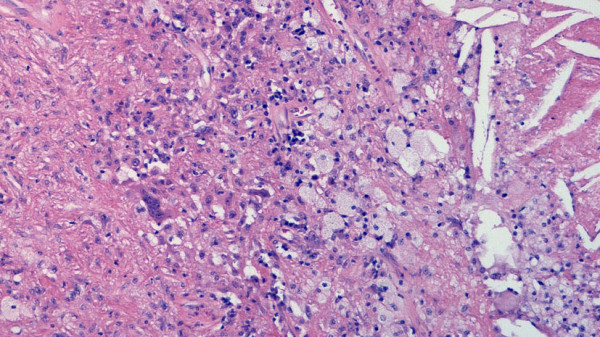
Histology of the resection specimen. Spindle tumor cells admixed with some multinucleated giant cells (left) and foamy macrophages (middle) and cholesterol clefts (right). (Hematoxyllin and Eosin x)

After reference to the literature on BFH, surgical en bloc resection was considered to be the appropriate treatment modality [2,3,12,13]. In accordance to the imaging results, the iliac crest could be preserved, while the linea terminalis of the left side had to be resected (Figure 3). Figure 4 shows the resected tumor specimen. Histological study of the resected specimen confirmed the diagnosis of BFH (Figure 5). The surgical margins were found to be free of tumor confirming complete tumor resection.

**Figure 6:**
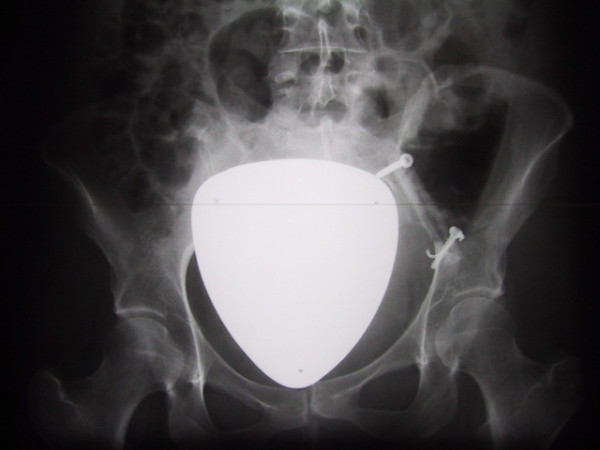
Status after interposition of the fibula transplant

The pelvic ring was then stabilized in a second operation using a non-vascularized fibular autograft that was impacted and stabilized with screws and a Kirschner-wire reconstructing the linea terminalis of the left pelvic ring (Figure 6). Post operatively she developed neurapraxia of the left lateral femoral cutaneous nerve, which resolved spontaneously within 6 months after surgery. Following two weeks of bed rest and limited restriction of movement up to 70° flexion of the hip joint, the patient was mobilized with partial weight bearing for three months.

**Figure 7:**
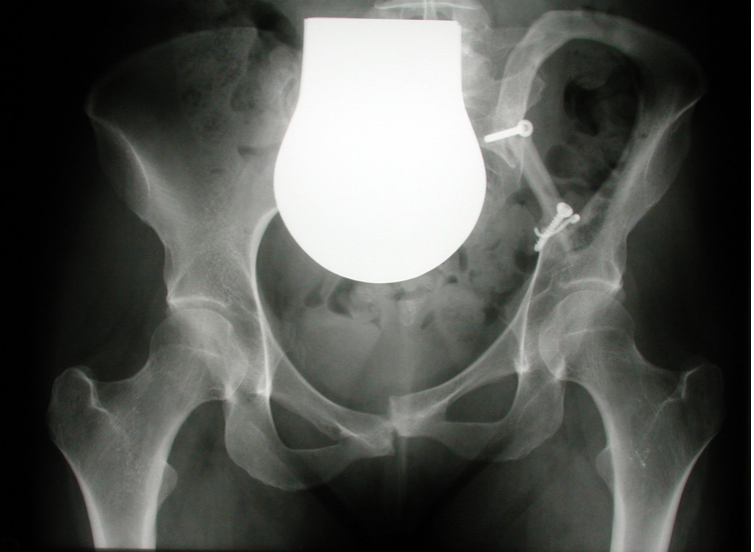
Radiographic check 18 months after surgery, showing identical position of the fibular transplant

Radiographic and MRI controls at 3, 6 and 12 months postoperatively showed no evidence of local recurrence or secondary dislocation of the reconstruction (Figure 7). A diastasis of the symphysis was noted, which we ascribe to the pregnancy one year prior to surgery. After 18 months of surgery the patient is free of disease and ambulates with full weight bearing.

## Discussion

A rare anatomical location of BFH accompanied by an extraordinary tumor size of 9.5 × 7 × 11 cm is presented in this report. To our knowledge, no BFH of the bone with a similar size has been published. Due to its size and location on both sides of the iliac wing, including a large osteolytic lesion of the *os ilium*, this tumor presented a challenge for surgical resection.

There are only a few studies addressing the stability of the pelvic ring following partial hemipelvectomy [14-17]. A variety of problems, such as persistent instability and complications caused by postoperative infections and transplant dislocation, have been reported [14-17]. The majority of the reconstructions reported have followed extensive resection of malignant bone tumors. Allograft [18], non-vascularized and vascularized auto grafts (fibula, tibia, and femur) [15,19,20], endoprosthetic replacement and other osteosynthetic procedures (i.e. with Kuntscher rods and K-wires or transpedicular and iliac screw systems) [14] have been used with differing degrees of success and variable clinical outcome. After total sacrectomy adequate reconstruction was reported in one study to have been achieved with transpedicular and iliac screw systems [21]. However K-wires were found to be insufficient for reconstruction of the ilium [14]. Within recent years, the use of biological transplants for such reconstructions has been reported in a number of cases with functionally satisfactory results [18,15,20].

In our case, we decided to reconstruct the inferior pelvic ring with an impacted non-vascularized fibular autograft to provide stability to the pelvic ring. No data is available on the necessity for reconstructing the linea terminalis when the continuity of the pelvic ring remains intact (a bony bridge between sacrum and ilium was left intact in our case). We are of the opinion that stabilization of the inner pelvic ring is necessary to prevent stress fracture of the remaining bone bridge.

After mobilization of the patient an increasing diastasis of the symphysis was noted (Figure 7). This might have been caused by moderate shortening of the implanted fibular graft, which can in turn result in rotation and tilting of the affected hemipelvis. In our case loosening of the symphysis following pregnancy and vaginal delivery prior to surgery too might have contributed to diastasis in our case.

Oncologically the marginal tumor resection has proved to be an adequate treatment. Despite high recurrence rates, especially in the case of large BFH [2], no local recurrence has so far been observed in our case. With a follow-up of 18 months after tumor resection as well as reconstruction of the pelvic ring it seems to become a successfully performed treatment for this patient.

## Competing interests

The author(s) declare that they have no competing interests.

## Authors' contributions

**PN **prepared the manuscript and participated in the operations.

**KL **carried out the radiographies and MR imaging and contributed the section on imaging

**MW **performed histological analysis and wrote pathology part of manuscript

**LB **and **DP **critically reviewed the manuscript for its scientific content and performed surgery.
